# Relationship between loneliness, social isolation and modifiable risk factors for cardiovascular disease: a latent class analysis

**DOI:** 10.1136/jech-2020-215539

**Published:** 2021-01-06

**Authors:** Feifei Bu, Andrew Steptoe, Daisy Fancourt

**Affiliations:** Department of Behavioural Science and Health, University College London, London, UK

**Keywords:** ageing, cardiovascular disease, cohort studies, health behaviour, social factors in

## Abstract

**Background:**

There is growing research into the effects of psychological and social factors such as loneliness and isolation on cardiovascular disease (CVD). However, it is unclear whether individuals with particular clusters of CVD risk factors are more strongly affected by loneliness and isolation. This study aimed to identify latent clustering of modifiable risk factors among adults aged 50+ and explore the relationship between loneliness, social isolation and risk factor patterns.

**Methods:**

Data from 8218 adults of English Longitudinal Study of Ageing were used in latent class analyses to identify latent classes of cardiovascular risk factors and predictors of class membership.

**Results:**

There were four latent classes: low-risk (30.2%), high-risk (15.0%), clinical-risk (42.6%) and lifestyle-risk (12.2%) classes. Loneliness was associated with a greater risk of being in the high-risk class (relative risk ratio (RRR) 2.40, 95% CI 2.40 to 1.96) and lifestyle-risk class (RRR 1.36, 95% CI 1.10 to 1.67) and a lower risk of being in the clinical-risk class (RRR 0.84, 95% CI 0.72 to 0.98) relative to the low-risk class. Social disengagement, living alone and low social contact were also differentially associated with latent class memberships.

**Conclusion:**

These findings supplement our existing knowledge of modifiable risk factors for CVD by showing how risk factors cluster together and how the risk patterns are related to social factors, offering important implications for clinical practice and preventive interventions.

## Introduction

Cardiovascular diseases (CVD) is a major health problem globally and in the UK. According to the British Heart Foundation,^1^ there are around 7.4 million people living with CVD in the UK. CVD is the second leading cause of death in the UK which accounted for 27% of all death.^2^ CVD, in particular stroke, is also a major contributor of acquired adult disability. It is reported that between 55% and 77% of stroke survivors are severely disabled or require assistance with activities of daily living.^3^ CVD imposes a major financial burden, costing the UK 9 billion for healthcare and another 4 billion for non-health care.^4^ The National Health Service (NHS) long-term plan has set CVD as one of its clinical priorities, setting an ambition to prevent CVD cases over the next 10 years.^5^

Previous research has identified a broad range of risk factors for CVD which can be classified into two groups: non-modifiable and modifiable risk factors. The former includes demographic characteristics (e.g., age, gender, ethnicity) and family history.^6^ The latter can be sorted into three domains, including: (1) clinical/biological risks such as obesity, diabetes, hypertension and dyslipidaemia,^7 8^ (2) psychosocial factors, such as stress, anxiety and depression^7 9^ and (3) behavioural/lifestyle risks, including sleep, drinking, smoking, diet and physical activity.^7 10^

Over the past two decades, there is growing research into social factors such as loneliness and social isolation. Several studies identified loneliness and social isolation as risk factors for CVD and CVD-specific mortality.^11^ Much research to date has suggested that these associations result from activation of a range of clinical risk mechanisms. For example, loneliness and social isolation were related to increased concentrations of stress hormones,^12^ blood pressure,^13^ levels of inflammatory markers^14^ and the risk of developing obesity, diabetes or hypertension.^15 16^ It was found that loneliness and social isolation exacerbated other psychosocial and behavioural risk factors such as higher levels of depression,^17^ decreased physical activity and increased smoking.^13^

However, while there are evidently multiple mechanisms by which social isolation and loneliness can affect CVD risks, it remains unclear if all individuals are equally affected. Given that risk factors tend to cluster together, it could be that certain groups of people, for example those who lead particularly unhealthy lifestyles having a number of behavioural risks or individuals with a number of clinical conditions, may be more strongly affected by loneliness and social isolation. This may not be manifested using the conventional risk score approach, which quantifies CVD risks but overlooks the pattern of combinations. Therefore, this study sought to identify clusters of modifiable CVD risk factors including clinical, psychosocial and behavioural risks using latent class analysis (LCA) and to explore the relationship between loneliness, social isolation and latent clustering of CVD risk factors.

## Data and method

Data came from the English Longitudinal Study of Ageing, a large-scale panel study of people aged 50 or over and their partners, living in private households in England. The original sample was drawn from participants from the Health Survey in England in 1998, 1999 and 2001. The first wave of data collection took place in 2002/2003, and participants have been followed biennially since. Data collection is carried out though face-to-face interviews, self-completion questionnaires and nurse visits (every 4 years). We used wave 4 (2008/2009) in this study because some variables of interest were not measured at earlier waves. Of 9886 core members in this wave, 8218 participants had a nurse visit to provide the data relevant to our analyses. Further analysis including covariates excluded participants with missing values, reducing the sample size to 5947.

### CVD risk indicators

Our analysis included risk indicators that were commonly used in risk assessment models such as the Systematic Coronary Risk Evaluation^18^ and Framingham Risk Score.^19^ These included hypertension, high cholesterol, diabetes, obesity and smoking. In addition, we also considered other indicators identified in the literature, such as depression, abnormal sleep, drinking, diet and physical activity.^7 10^

Hypertension was defined as having a diagnosis or a systolic blood pressure ≥140 mm Hg or diastolic blood pressure ≥90 mm Hg.^20^ Cholesterol risk was derived based on total cholesterol to high-density lipoprotein (HDL) cholesterol (TC/HDL-C) ratio, >4 for men and >3.5 for women.^21^ Diabetes was defined as having a diagnosis or a fasting glucose level ≥7 mmol/L.^22^ Abdominal obesity was defined as having a waist circumference ≥102 cm for men and ≥88 cm for women.^23^

Depression was measured using the eight-item Centre for Epidemiological Studies-Depression (CES-D) Scale, in addition to diagnoses of depression. The cut-off point 4 of the CES-D scale was used for defining serious depressive symptoms.^24^

Both long and short sleep durations have been associated with CVD.^25^ We defined disturbed sleep as ≤5 hours or ≥9 hours.^26^ Smoking was coded as a binary variable indicating whether participants were current smokers. Heavy drinking was defined as if participants drank daily and had ≥14 units of alcoholic drink in the past week. Poor diet was defined as having ≤5 portions of fruits and vegetables daily. Physical inactivity was defined as exercising less than weekly at either vigorous (eg, running, swimming, cycling, aerobics etc.) or moderate levels (eg, gardening, walking etc.).

Most of these risk variables were positively associated with each other as expected (see online supplemental figure S1). However, heavy drinking was negatively associated with many other risks. It was possible that drinking was confounded with social factors, for example, the frequency of social interactions. Therefore, drinking was excluded from the analyses.

10.1136/jech-2020-215539.supp1 Supplementary data

### Loneliness and social isolation

Loneliness was measured using the three-item University of California, Los Angeles (UCLA) loneliness scale. Social isolation was measured in three ways. Living alone was coded as a binary variable to capture domestic isolation. Low social contact was derived from the frequency of social contacts (meeting up or speaking on the phone) with children, relatives and friends (a six-point scale from three or more times a week to never). Finally, social disengagement was measured by the frequency of (1) group membership (none, one group or two or more groups), (2) formal volunteering (a five-point scale from never to twice a month or more) and (3) engagement with cultural activities, for example, going to museums and exhibitions (a six-point scale from never to twice a month or more). Both low social contact and social disengagement were generated by confirmatory factor analysis (CFA) using the mean-adjusted and variance-adjusted weighted least squares (WLSMV) estimator. Both were standardised to a mean of 0 and standard deviation (SD) of 1, with higher scores indicating less social contact and increased social disengagement.

### Other covariates

In addition to modifiable risk factors in the LCA model, our analyses also included non-modifiable risk factors, including gender (women vs men), ethnicity (white vs non-white) and age groups (50–59, 60–69, 70–79, 80+). There were also a set of socioeconomic measures, including education (degree or above, a level or below, no qualification), social class recoded from the National Statistics Socio-Economic Classification (high, medium, low) and household wealth in deciles. Finally, we considered existing CVD diagnoses, a binary variable indicating whether participants had reported any of the following diagnoses: angina, heart attack, congestive heart failure, heart murmur, abnormal heart rhythm, stroke and other heart disease.

### Statistical analysis

We used LCA to depict the clustering of modifiable CVD risks. LCA posits that there is an underlying latent structure that divides a population into mutually exclusive and exhaustive classes. To determine the number of classes, we compared model fits on the basis of Akaike information criterion (AIC) and Bayesian information criterion (BIC). For both indices, smaller values indicate a finer balance between model fit and parsimony. We started with an unconditional LCA model, including only modifiable risk factors. We then introduced loneliness and social isolation measures as predictors of the latent class membership (model I). We then further added socioeconomic measures and existing CVD conditions (model II). These were estimated based on a multinomial logistic regression implemented simultaneously with the LCA model. CFA scores with WLSMV estimators were generated in R 3.5.1, but the full analyses were carried out using Stata V.15.

## Results

### Descriptive

Of 8218 participants without missing values in any of the CVD risk indicators, 51% had hypertension, 55% with high cholesterol, 9% diabetes, 54% abdominal obesity, 17% depression. Approximately 6% of participants experienced disturbed sleep. About 12% of them were current smokers, 43% not having a healthy diet and 34% being physically inactive. For sample demographic characteristics, see online supplemental table S1.

### Latent classes

**Figure 1:**
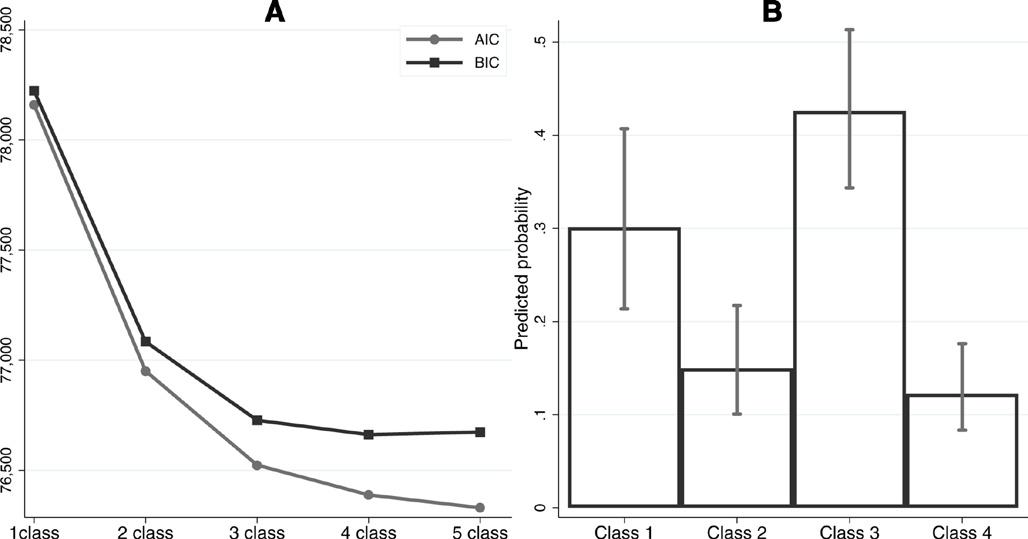
Model fit indices and predicted probability of class membership. AIC, Akaike information criterion; BIC, Bayesian information criterion.

**Figure 2:**
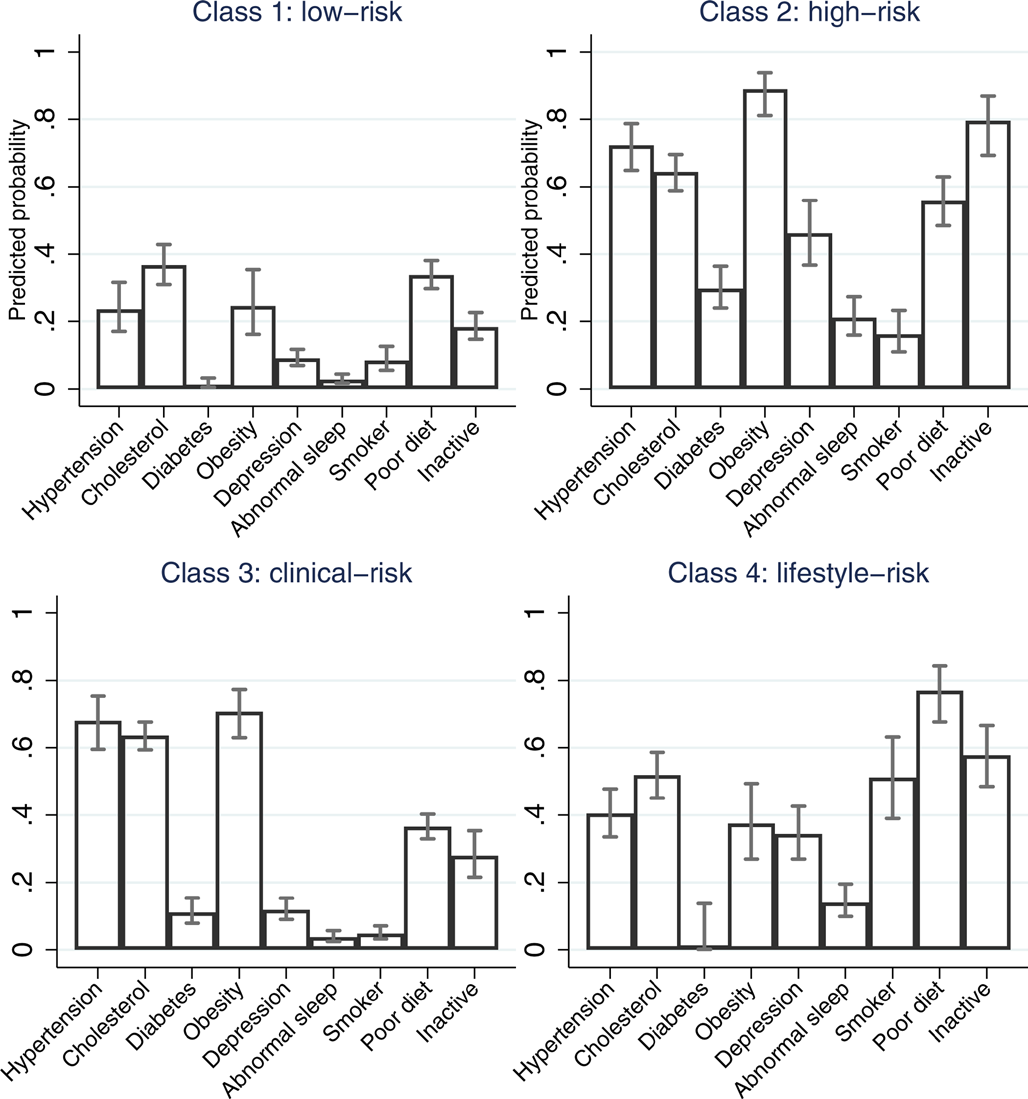
Predicted probabilities of cardiovascular disease risks by latent classes from latent class analysis.

Starting with a single-class model, additional classes improved the model fit up to a four-class specification (figure 1A). BIC marginally increased from the four-class to five-class model, so the four-class model was identified as the optimal solution. As shown in figure 1B, the largest was class 3 (about 42.6%), followed by class 1 (30.2%), class 2 (15.0%) and class 4 (12.2%). The predicted probabilities of risk indicators for each class are shown in figure 2. Class 1 was termed the ‘low-risk’ group which consisted of people with a healthy lifestyle and a very low probability of having clinical risks. Class 2 contained people with a very high probability of having both clinical and behavioural risks (in particular, poor diet and physical inactivity). It was labelled as the ‘high-risk’ group. Class 3 which was characterised as having a high probability of clinical risks but a relatively healthy lifestyle was labelled as the ‘clinical-risk’ group. Finally, class 4 was characterised as having an unhealthy lifestyle, but with only a moderate probability of having clinical risks. This was labelled as the ‘lifestyle-risk’ group.

### Loneliness, isolation and class membership

Table 1 reports the estimated relative risk ratio (RRR) and 95% CI for loneliness and social isolation measures using the low-risk class as reference. In the full model, loneliness was especially associated with the probability of being in the high-risk class (model II: RRR 2.40, 95% CI 2.40 to 1.96) and the lifestyle-risk class (model II: RRR 1.36, 95% CI 1.10 to 1.67) relative to the low-risk class. People who were lonely were less likely to be in the clinical-risk class (model II: RRR 0.84, 95% CI 0.72 to 0.98). Living alone was associated with higher risk of being in all three classes than the low-risk class, but when additionally controlling for socioeconomic factors and existing CVD conditions, only the association with being in the lifestyle-risk class remained (model II: RRR 1.86, 95% CI 1.18 to 2.91). Low social contact was only associated with a lower risk of being in the high-risk class (model II: RRR 0.78, 95% CI 0.66 to 0.92). Finally, social disengagement was associated with a higher risk of being in the lifestyle-risk class especially (model II: RRR 3.70, 95% CI 2.91 to 4.70), as well as the high-risk class (model II: RRR 3.03, 95% CI 2.40 to 3.83) and the clinical-risk class (model II: RRR 1.22, 95% CI 1.04 to 1.43).

**Table 1:** Relative risk ratio (RRR) and 95% CI from the latent class analysis model with covariates using the low-risk group as the reference (N=5947)

	Class 2: high-risk (vs low-risk)			Class 3: clinical-risk (vs low-risk)			Class 4: lifestyle-risk (vs low-risk)		
	RRR	95% CI	P value	RRR	95% CI	P value	RRR	95% CI	P value
Model I(controlling for age, gender, ethnicity)									
Loneliness	**2.44**	**2.02 to 2.96**	**0.014**	**0.83**	**0.71 to 0.96**	**<0.001**	**1.45**	**1.19 to 1.78**	**<0.001**
Living alone	**1.77**	**1.20 to 2.60**	**0.037**	**1.39**	**1.02 to 1.89**	**0.004**	**3.11**	**2.06 to 4.67**	**<0.001**
Low social contact	0.71	0.61 to 0.82	0.283	**0.94**	**0.84 to 1.05**	**<0.001**	1.00	0.84 to 1.18	0.994
Social disengagement	**4.25**	**3.21 to 5.63**	**0.005**	**1.28**	**1.08 to 1.53**	**<0.001**	**5.77**	**4.51 to 7.37**	**<0.001**
Model II (model I+SES+existing CVD conditions)									
Loneliness	**2.40**	**1.96 to 2.94**	**<0.001**	**0.84**	**0.72 to 0.98**	**0.025**	**1.36**	**1.10 to 1.67**	**0.004**
Living alone	1.15	0.77 to 1.72	0.484	1.19	0.87 to 1.65	0.276	**1.86**	**1.18 to 2.91**	**0.007**
Low social contact	**0.78**	**0.66 to 0.92**	**0.003**	0.95	0.84 to 1.07	0.425	1.13	0.94 to 1.35	0.192
Social disengagement	**3.03**	**2.40 to 3.83**	**<0.001**	**1.22**	**1.04 to 1.43**	**0.017**	**3.70**	**2.91 to 4.70**	**<0.001**

**Figure 3:**
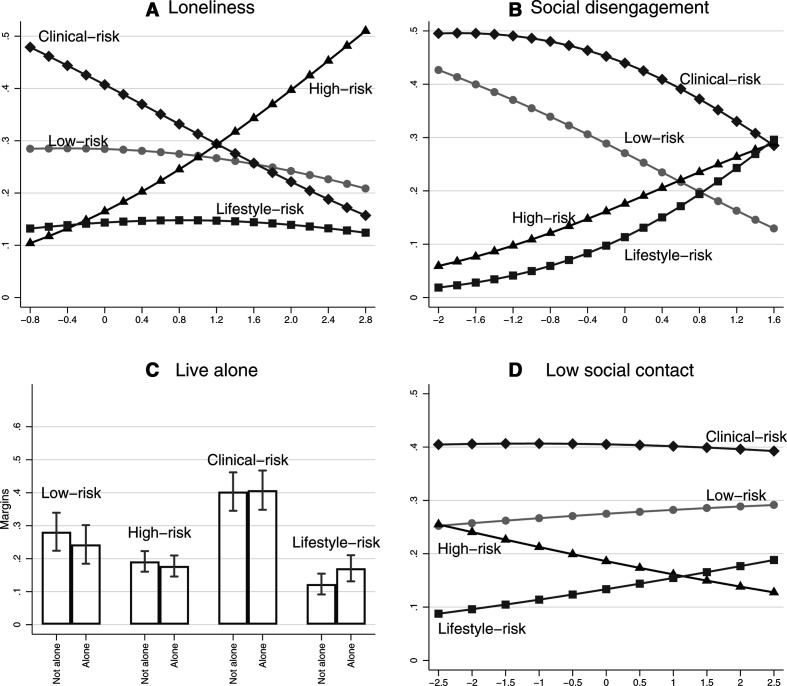
Predicted probabilities of latent cardiovascular disease risk groups.

Figure 3 presents the predicted probability of class membership by loneliness and social isolation from the full model. Higher levels of loneliness were markedly associated with increased probability of being in the high-risk class and lowered probability of being in the clinical-risk class as well as a slight decrease of being in the low-risk class (figure 3A). Greater social disengagement was associated with declines in the probability of being in the low-risk or clinical-risk classes and increased probability of being in the lifestyle-risk and high-risk classes (figure 3B). Living alone was associated with a lower probability of being in the low-risk class and a higher probability of being in the lifestyle-risk class (figure 3C). Finally, lower social contact was associated with a slight decrease in probability of being in the high-risk class and a slight increase of being in the lifestyle-risk class (non-significant) (figure 3D).

As a sensitivity analysis, we also fitted a multigroup latent class model by gender. No evidence was found that the latent structure of CVD risks differed between men and women (see online supplemental figures S2 and S3).

## Discussion

Our analyses identified four latent classes of modifiable CVD risks among older adults: (1) a low-risk class with few risks, (2) high-risk class with a range of risk factors, (3) clinical-risk class, who have poor health but a healthy lifestyle and (4) lifestyle-risk class, who have an unhealthy lifestyle but relatively good health. Further, we found that social disengagement was consistently associated with being in one of the higher-risk classes (classes 2–4), while other social factors, such as loneliness, living alone and low social contact had differential associations with different patterns of modifiable risks.

We found that modifiable CVD risk factors tend to cluster around two groups: clinical and lifestyle risks. As a result, people with hypertension have a high probability of having other CVD-related clinical conditions; and poor diet is likely to be companied by other lifestyle risks, such as smoking and physical inactivity. This echoes previous studies that have shown a high prevalence of concomitant conditions, such as diabetes, hypertension, dyslipidaemia and obesity,^27^ and the clustering of health behaviours within individuals.^28^ No class exhibits a mixture of high and low lifestyle or clinical risks. Moreover, we found that depression tend to cooccur specifically with lifestyle risks. This is supported by evidence showing a strong association between lifestyle measures and depression.^29^ Notably, depression was not characteristic of the clinical-risk class. While depression is often comorbid with conditions such as hypertension, diabetes and obesity,^30–32^ our results suggest two possibilities. Either among individuals with these physical health conditions, an absence of depression might help to buffer against the risk of engaging in unhealthy behaviours,^33^ or the engagement in a healthy lifestyle might help to reduce the risk of developing depression.^29^

Our findings build on previous literature suggesting that social factors are associated with CVD risk, but extends these findings by showing differential associations with different patterns of risk factors. Our most consistent finding was that social disengagement was associated with a heightened probability of being in any of the classes other than the low-risk class. Social engagement in activities such as volunteering, community groups and culture has been shown to build aspects of social capital and enhance individual’s informational and structural resources.^34^ Therefore, it is possible that individuals who are disengaged have less access to such support and are more likely to lead unhealthy lifestyles and accumulate avoidable health conditions. Importantly, this finding persisted independent of wealth, social class and education, suggesting it does not merely reflect socioeconomic factors. Further, the association was less strong for the clinical-risk group, who may already be engaging more proactively in healthy behaviours.

We also found that loneliness was associated with an increased probability of being in the high-risk class but a lowering probability of being in the clinical-risk class. The high-risk versus clinical-risk class is differentiated by the fact that the former contains individuals with depression who are engaging in unhealthy behaviours while the clinical-risk group contains individuals who have poor health but a healthy lifestyle and little evidence of depression. This is consistent with previous research showing that individuals who are lonely are more likely to be depressed and engage in unhealthy behaviours.^35^ However, it is notable that loneliness is associated with a lower risk of being in the clinical-risk group compared with the low-risk group. Levels of depression were similar across these two classes, such that the only major difference is that the clinical-risk group has poor health conditions. One potential explanation for this finding could be that people from the clinical-risk group may have engaged in more healthy behaviours as a result of their health conditions; a behavioural change that could have caused them to consider their lives more broadly and led them to address any deficits in the quality of their social interactions. However, this remains to be tested further.

Our third finding was that living alone and low social contact only had small associations with class membership. Living alone was associated with a higher probability of being in the lifestyle-risk group, which could be due to the absence of another to help modify unhealthy behaviours.^36^ Indeed, it has previously been shown that people are more likely to make a positive health behaviour change if their partner does too.^37^ But any other associations for living alone were attenuated when accounting for socioeconomic factors. Further, low social contact was associated with a lower probability of being in the high-risk class. It is possible that unhealthy behaviours among individuals with existing health conditions may be partly driven by socialising.^38^ Conversely, among individuals who have fewer health conditions (the lifestyle-risk group) or are being more proactive in their health behaviours (the clinical-risk group), social contact does not present as a vulnerability. It is also relevant to draw parallels between the finding for low social contact and social disengagement and the probability of being in the high-risk group. Low social contact presents a decreased risk of being high risk, while engagement with community activities has protective associations. This builds on findings from studies showing that engagement in community activities can help to build factors such as self-esteem, self-efficacy and agency that may support health behaviours.^39^

To our knowledge, our study is the first to investigate the latent structure of modifiable cardiovascular risks in a western context. It has the advantage of a large and representative sample. Moreover, our study has shown the link between social factors and the latent structure of cardiovascular risks for the first time. However, the study is not without limitations. First, our list of risk indicators is by no means exhaustive. Due to data constraint, we were unable to consider other risk factors such as stress. Further, as our analysis is based on people aged 50 or over, it remains unknown whether the same pattern occurs in younger age groups. Finally, this is a cross-sectional study, so causality cannot be established and our results must be interpreted with caution. Future research could explore how clusters of CVD risk factors evolve over time and which changing behavioural or clinical risk patterns pose the highest risk for CVD.

Overall, these findings supplement our existing knowledge of modifiable CVD risk factors by showing how different risks cluster together, and shows for the first time the differential patterns of association with different social factors. This study has implications for clinical practice, where an understanding of risk patterns could support the development of interventions for different groups of people, offering tailored health recommendation protocols.

**What is already known on this subject**

- Previous research has established a range of cardiovascular disease (CVD) risk factors. There has been evidence for the association between social factors, such as loneliness and social isolation, and CVD events and risks.

**What this study adds**

- This study has shown for the first time the latent structure of the modifiable cardiovascular disease (CVD) risk factors in a Western context. Further, it shows how loneliness and different aspects of social isolation are differentially associated with the latent classes of CVD risk factors. Our findings offer important implications for clinical practice and preventive interventions.

## Data Availability

Data are available in a public, open access repository. Data from ELSA are available from the UK Data Service (https://ukdataservice.ac.uk/). The access to the linked data with Hospital Episode Statistics can be obtained from NatCen.

## References

[1] British Heart Foundation . Uk Factsheet, 2019.

[2] Townsend N , Bhatnagar P , Wilkins E . Cardiovascular disease statistics London British heart Foundation, 2015.

[3] Feigin VL , Lawes CMM , Bennett DA , et al . Stroke epidemiology: a review of population-based studies of incidence, prevalence, and case-fatality in the late 20th century. Lancet Neurol 2003;2:43–53. 10.1016/S1474-4422(03)00266-7 12849300

[4] Wilkins E , Wilson L , Wickramasinghe K . European cardiovascular disease statistics 2017. Brussels: European Heart Network, 2017.

[5] NHS . The NHS long term plan, 2019.

[6] Hippisley-Cox J , Coupland C , Vinogradova Y , et al . Predicting cardiovascular risk in England and Wales: prospective derivation and validation of QRISK2. BMJ 2008;336:1475–82. 10.1136/bmj.39609.449676.25 18573856 PMC2440904

[7] Yusuf S , Hawken S , Ôunpuu S , et al . Effect of potentially modifiable risk factors associated with myocardial infarction in 52 countries (the INTERHEART study): case-control study. Lancet 2004;364:937–52. 10.1016/S0140-6736(04)17018-9 15364185

[8] Huxley R , Mendis S , Zheleznyakov E , et al . Body mass index, waist circumference and waist:hip ratio as predictors of cardiovascular risk--a review of the literature. Eur J Clin Nutr 2010;64:16–22. 10.1038/ejcn.2009.68 19654593

[9] Steptoe A , Kivimäki M , Stress KM . Stress and cardiovascular disease: an update on current knowledge. Annu Rev Public Health 2013;34:337–54. 10.1146/annurev-publhealth-031912-114452 23297662

[10] Timmis A , Townsend N , Gale C , et al . European Society of cardiology: cardiovascular disease statistics 2017. Eur Heart J 2018;39:508–79. 10.1093/eurheartj/ehx628 29190377

[11] Valtorta NK , Kanaan M , Gilbody S , et al . Loneliness and social isolation as risk factors for coronary heart disease and stroke: systematic review and meta-analysis of longitudinal observational studies. Heart 2016;102:1009–16. 10.1136/heartjnl-2015-308790 27091846 PMC4941172

[12] Hackett RA , Hamer M , Endrighi R , et al . Loneliness and stress-related inflammatory and neuroendocrine responses in older men and women. Psychoneuroendocrinology 2012;37:1801–9. 10.1016/j.psyneuen.2012.03.016 22503139

[13] Shankar A , McMunn A , Banks J , et al . Loneliness, social isolation, and behavioral and biological health indicators in older adults. Health Psychol 2011;30:377–85. 10.1037/a0022826 21534675

[14] Walker E , Ploubidis G , Fancourt D . Social engagement and loneliness are differentially associated with neuro-immune markers in older age: time-varying associations from the English longitudinal study of ageing. Brain Behav Immun 2019;82:224–9. 10.1016/j.bbi.2019.08.189 31491488 PMC6997881

[15] Whisman MA . Loneliness and the metabolic syndrome in a population-based sample of middle-aged and older adults. Health Psychol 2010;29:550–4. 10.1037/a0020760 20836610

[16] Momtaz YA , Hamid TA , Yusoff S , et al . Loneliness as a risk factor for hypertension in later life. J Aging Health 2012;24:696–710. 10.1177/0898264311431305 22422758

[17] Cacioppo JT , Hawkley LC , Thisted RA . Perceived social isolation makes me sad: 5-year cross-lagged analyses of loneliness and depressive symptomatology in the Chicago health, aging, and social relations study. Psychol Aging 2010;25:453–63. 10.1037/a0017216 20545429 PMC2922929

[18] Conroy RM , Pyörälä K , Fitzgerald AP , et al . Estimation of ten-year risk of fatal cardiovascular disease in Europe: the score project. Eur Heart J 2003;24:987–1003. 10.1016/S0195-668X(03)00114-3 12788299

[19] D'Agostino RB , Vasan RS , Pencina MJ , et al . General cardiovascular risk profile for use in primary care: the Framingham heart study. Circulation 2008;117:743–53. 10.1161/CIRCULATIONAHA.107.699579 18212285

[20] Mancia G , Sega R , Milesi C , et al . Blood-Pressure control in the hypertensive population. The Lancet 1997;349:454–7. 10.1016/S0140-6736(96)07099-7 9040574

[21] Millán J , Pintó X , Muñoz A , et al . Lipoprotein ratios: physiological significance and clinical usefulness in cardiovascular prevention. Vasc Health Risk Manag 2009;5:757–65. 19774217 PMC2747394

[22] Expert Committee on the Diagnosis and Classification of Diabetes Mellitus . Report of the expert Committee on the diagnosis and classification of diabetes mellitus. Diabetes Care 2003;26 Suppl 1:S5–20. 10.2337/diacare.26.2007.S5 12502614

[23] McGee S . Evidence-Based physical diagnosis. 4th Edn. Philadelphia: Elsevier, 2018.

[24] Han B , Symptoms D . Depressive symptoms and self-rated health in community-dwelling older adults: a longitudinal study. J Am Geriatr Soc 2002;50:1549–56. 10.1046/j.1532-5415.2002.50411.x 12383153

[25] Cappuccio FP , Cooper D , D'Elia L , et al . Sleep duration predicts cardiovascular outcomes: a systematic review and meta-analysis of prospective studies. Eur Heart J 2011;32:1484–92. 10.1093/eurheartj/ehr007 21300732

[26] Min Y , Slattum PW . Poor sleep and risk of falls in community-dwelling older adults: a systematic review. J Appl Gerontol 2018;37:1059–84. 10.1177/0733464816681149 28380704

[27] Weycker D , Nichols GA , O'Keeffe-Rosetti M , et al . Risk-Factor clustering and cardiovascular disease risk in hypertensive patients. Am J Hypertens 2007;20:599–607. 10.1016/j.amjhyper.2006.10.013 17531915

[28] Poortinga W . The prevalence and clustering of four major lifestyle risk factors in an English adult population. Prev Med 2007;44:124–8. 10.1016/j.ypmed.2006.10.006 17157369

[29] Lopresti AL , Hood SD , Drummond PD . A review of lifestyle factors that contribute to important pathways associated with major depression: diet, sleep and exercise. J Affect Disord 2013;148:12–27. 10.1016/j.jad.2013.01.014 23415826

[30] Meng L , Chen D , Yang Y , et al . Depression increases the risk of hypertension incidence: a meta-analysis of prospective cohort studies. J Hypertens 2012;30:842–51. 10.1097/HJH.0b013e32835080b7 22343537

[31] Yu M , Zhang X , Lu F , et al . Depression and risk for diabetes: a meta-analysis. Can J Diabetes 2015;39:266–72. 10.1016/j.jcjd.2014.11.006 25773933

[32] Luppino FS , de Wit LM , Bouvy PF , et al . Overweight, obesity, and depression: a systematic review and meta-analysis of longitudinal studies. Arch Gen Psychiatry 2010;67:220–9. 10.1001/archgenpsychiatry.2010.2 20194822

[33] Strine TW , Mokdad AH , Dube SR , et al . The association of depression and anxiety with obesity and unhealthy behaviors among community-dwelling us adults. Gen Hosp Psychiatry 2008;30:127–37. 10.1016/j.genhosppsych.2007.12.008 18291294

[34] Guillen L , Coromina L , Saris WE . Measurement of social participation and its place in social capital theory. Soc Indic Res 2011;100:331–50. 10.1007/s11205-010-9631-6

[35] Mezuk B , Choi M , DeSantis AS , et al . Loneliness, depression, and inflammation: evidence from the multi-ethnic study of atherosclerosis. PLoS One 2016;11:e0158056–e56. 10.1371/journal.pone.0158056 27367428 PMC4930171

[36] Umberson D , Crosnoe R , Reczek C . Social relationships and health behavior across life course. Annu Rev Sociol 2010;36:139–57. 10.1146/annurev-soc-070308-120011 21921974 PMC3171805

[37] Jackson SE , Steptoe A , Wardle J . The influence of partner's behavior on health behavior change: the English longitudinal study of ageing. JAMA Intern Med 2015;175:385–92. 10.1001/jamainternmed.2014.7554 25599511

[38] Cruz JE , Emery RE , Turkheimer E . Peer network drinking predicts increased alcohol use from adolescence to early adulthood after controlling for genetic and shared environmental selection. Dev Psychol 2012;48:1390–402. 10.1037/a0027515 22390657 PMC3616641

[39] Adams KB , Leibbrandt S , Moon H . A critical review of the literature on social and leisure activity and wellbeing in later life. Ageing Soc 2011;31:683–712. 10.1017/S0144686X10001091

