# Supplementary material for: Relationship between loneliness, social isolation and modifiable risk factors for cardiovascular disease: a latent class analysis
Source: J Epidemiol Community Health. 2021 Jan 6;75(8):749–54. doi: 10.1136/jech-2020-215539 (PMC8292586; doi:10.1136/jech-2020-215539)
Supplement: Supplementary data [file jech-2020-215539supp001.pdf]

Supplementary Material

	hypertension	Diabetes	Cholesterol	Obesity	Depression	Ab sleep	Smoking	Drinking	Poor diet
Diabetes	0.46								
Cholesterol	0.22	0.17							
Obesity	0.37	0.49	0.28						
Depression	0.14	0.23	0.06	0.21					
Ab sleep	0.08	0.25	0.16	0.21	0.55				
Smoking	-0.14	-0.08	0.09	-0.13	0.32	0.26			
Drinking	0.07	-0.13	-0.21	-0.07	-0.26	-0.26	-0.02		
Poor diet	0.05	0.03	0.06	-0.00	0.14	0.10	0.43	0.08	
Inactive	0.17	0.37	0.04	0.27	0.4	0.42	0.29	-0.21	0.24

p<0.001 (chi^2 test)

p<0.01 (chi^2 test)

p<0.05 (chi^2 test)

Fig S1. Correlation matrix of the CVD risk variables (Goodman and Kruskal's gamma)

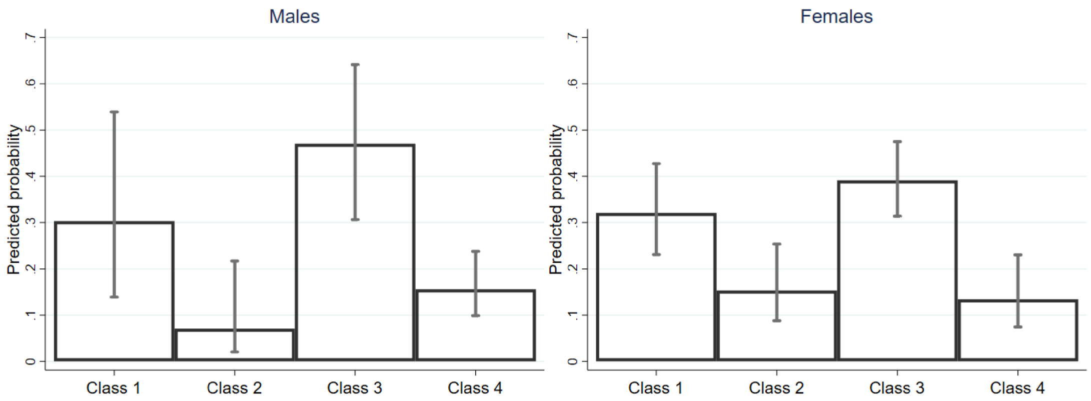

Fig S2. Predicted probability of class membership by sex from the multi-group model

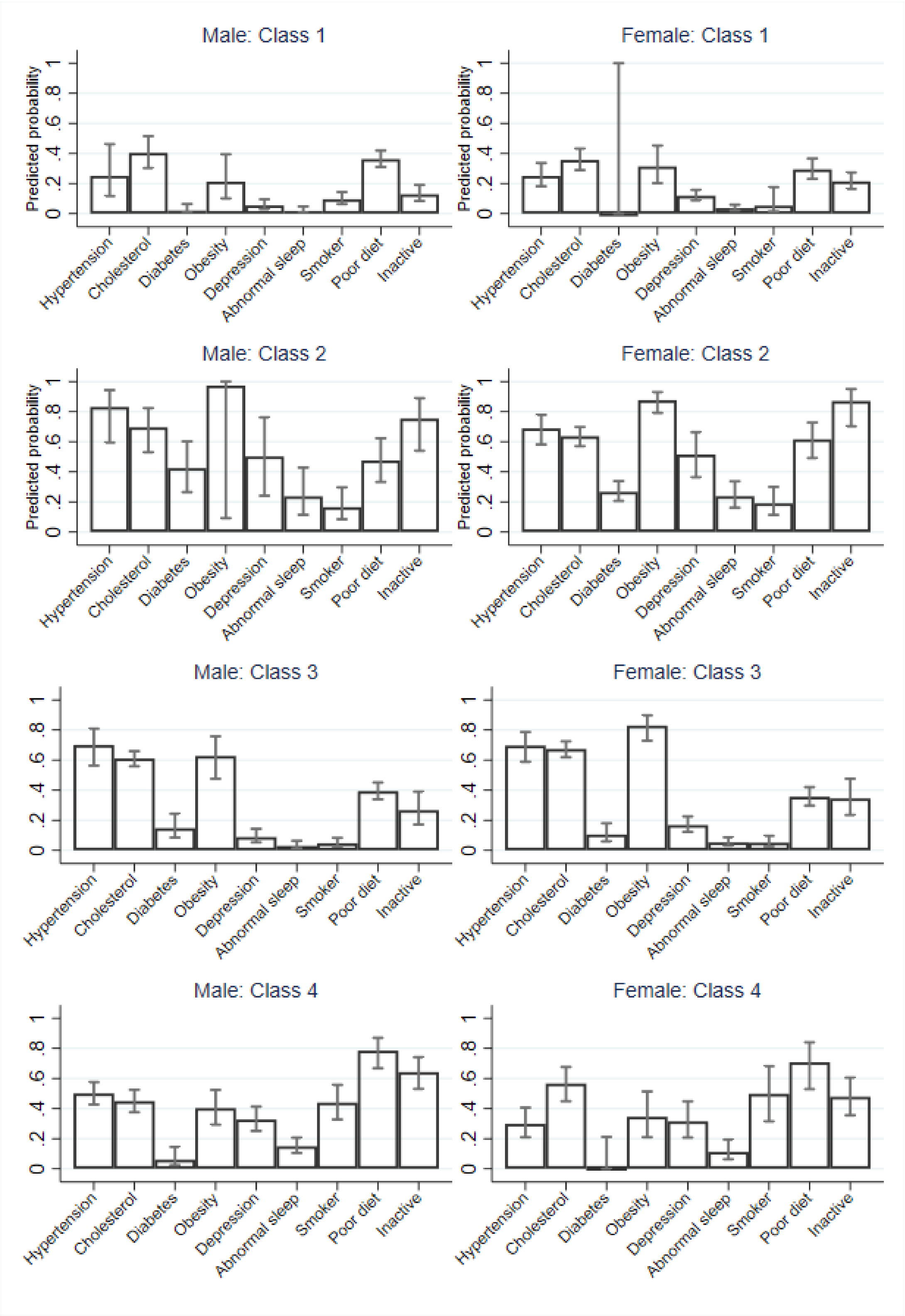

Fig S3. Predicted probabilities of CVD risks by latent classes from LCA and sex

Supplementary Table S1: Participants' characteristics by latent classes

		All %	Class 1: Low-risk	Class 2: High-risk	Class 3: Clinical-risk	Class 4: Lifestyle-risk
Gender:	Women	55.05	52.21	64.91	54.11	56.94
Ethnicity:	Non-white	2.56	2.12	4.26	2.50	2.12
Age:	50-59	28.26	36.14	19.47	23.72	36.69
	60-69	37.11	38.22	33.27	37.73	34.99
	70-79	24.43	18.72	28.30	28.10	19.69
	80+	10.20	6.92	18.97	10.45	8.64
Education:	Low	28.01	18.37	46.39	27.52	40.65
	Medium	40.40	40.39	35.71	41.62	40.23
	High	31.58	41.24	17.90	30.86	19.12
Social class:	Low	30.38	23.13	45.11	29.19	43.13
	Medium	35.36	35.15	33.37	35.82	36.32
	High	34.26	41.71	21.52	34.99	20.55
Wealth:	20% lowest	16.67	9.57	34.61	13.57	34.82
	Medium	60.66	58.96	55.79	63.77	56.50
	20% highest	22.67	31.47	9.61	22.66	8.67
Loneliness:	20% highest	20.23	16.23	38.88	16.61	33.16
Living:	Alone	25.42	19.76	40.97	23.34	36.12
Social isolation:	20% highest	19.96	19.07	19.49	20.10	23.45
Disengagement:	20% highest	23.62	13.45	46.16	21.33	49.34
Health:	Existing CVD	24.47	17.57	40.67	25.43	21.95
N		8,218†	2,601	986	3,925	706

Notes: † Education (0.2%), social class (2.2%), wealth (6.3%), loneliness (10.9%), social isolation (20.0%) and social disengagement (16.8%) contained missing values.

Bu F, *et al. J Epidemiol Community Health* 2021; 75:749–754. doi: 10.1136/jech-2020-215539
